# Supplementary material for: Biology of the mRNA Splicing Machinery and Its Dysregulation in Cancer Providing Therapeutic Opportunities
Source: Int J Mol Sci. 2021 May 12;22(10):5110. doi: 10.3390/ijms22105110 (PMC8150589; doi:10.3390/ijms22105110)
Supplement: Supplementary file 1 [file ijms-22-05110-s001.zip › ijms-1192958-supplementary.pdf]

Table S1. Overview of spliceosomal proteins according to KEGG, amiGO and Reactome databases.

Gene	Category	Gene ID
NCBP1	Cap binding complex	4686
NCBP2	Cap binding complex	22916
NCBP2L	Cap binding complex	392517
BUB3	Complex A specific	9184
CDK11A	Complex A specific	728642
CDK11B	Complex A specific	984
FUS	Complex A specific	2521
HTATSF1	Complex A specific	27336
RBM10	Complex A specific	8241
RBM20	Complex A specific	282996
RBM5	Complex A specific	10181
SF1	Complex A specific	7536
SUGP1	Complex A specific	57794
TET1	Complex A specific	80312
IK	Complex B specific	3550
MFAP1	Complex B specific	4236
SMU1	Complex B specific	55234
THRAP3	Complex B specific	9967
UBL5	Complex B specific	59286
CCDC130	Complex C specific	81576
DDX41	Complex C specific	51428
DGCR14	Complex C specific	8220
DHX35	Complex C specific	60625
FAM32A	Complex C specific	26017
FAM50A	Complex C specific	9130
FAM50B	Complex C specific	26240
FRA10AC1	Complex C specific	118924
FRG1	Complex C specific	2483
GPATCH1	Complex C specific	55094
NOSIP	Complex C specific	51070
PABPC1	Complex C specific	26986
PABPC1L	Complex C specific	80336
PABPC1L2A	Complex C specific	340529
PABPC1L2B	Complex C specific	645974
PABPC3	Complex C specific	5042
PABPC4	Complex C specific	8761
PABPC4L	Complex C specific	132430
PABPC5	Complex C specific	140886
RNF113A	Complex C specific	7737
TFIP11	Complex C specific	24144
WDR83	Complex C specific	84292
ZCCHC8	Complex C specific	55596
ACIN1	EJC/TREX	22985
ALYREF	EJC/TREX	10189
DDX39B	EJC/TREX	7919
EIF4A3	EJC/TREX	9775
MAGOH	EJC/TREX	4116

MAGOHB	EJC/TREX	55110
PNN	EJC/TREX	5411
RBM8A	EJC/TREX	9939
THOC1	EJC/TREX	9984
THOC2	EJC/TREX	57187
THOC3	EJC/TREX	84321
BCLAF1	General mRNP proteins	9774
ELAVL1	General mRNP proteins	1994
ILF2	General mRNP proteins	3608
ILF3	General mRNP proteins	3609
RBM23	General mRNP proteins	55147
RBM39	General mRNP proteins	9584
YBX1	General mRNP proteins	4904
ZC3H18	General mRNP proteins	124245
HNRNPA0	hnRNP proteins	10949
HNRNPA1	hnRNP proteins	3178
HNRNPA1L2	hnRNP proteins	144983
HNRNPA2B1	hnRNP proteins	3181
HNRNPA3	hnRNP proteins	220988
HNRNPAB	hnRNP proteins	3182
HNRNPC	hnRNP proteins	3183
HNRNPCL1	hnRNP proteins	343069
HNRNPD	hnRNP proteins	3184
HNRNPDL	hnRNP proteins	9987
HNRNPF	hnRNP proteins	3185
HNRNPH1	hnRNP proteins	3187
HNRNPH2	hnRNP proteins	3188
HNRNPH3	hnRNP proteins	3189
HNRNPK	hnRNP proteins	3190
HNRNPL	hnRNP proteins	3191
HNRNPLL	hnRNP proteins	92906
HNRNPM	hnRNP proteins	4670
HNRNPR	hnRNP proteins	10236
HNRNPU	hnRNP proteins	3192
HNRNPUL1	hnRNP proteins	11100
HNRNPUL2	hnRNP proteins	221092
MSL1	hnRNP proteins	339287
MSL2	hnRNP proteins	55167
PCBP1	hnRNP proteins	5093
PCBP2	hnRNP proteins	5094
RALY	hnRNP proteins	22913
RBMX	hnRNP proteins	27316
RBMXL1	hnRNP proteins	494115
RBMXL2	hnRNP proteins	27288
RBMXL3	hnRNP proteins	139804
SYNCRIP	hnRNP proteins	10492
CPSF3L	Integrator complex	54973
INTS1	Integrator complex	26173
INTS10	Integrator complex	55174
INTS12	Integrator complex	57117

INTS2	Integrator complex	57508
INTS3	Integrator complex	65123
INTS4	Integrator complex	92105
INTS5	Integrator complex	80789
INTS6	Integrator complex	26512
INTS7	Integrator complex	25896
INTS8	Integrator complex	55656
INTS9	Integrator complex	55756
LSM2	LSm proteins	57819
LSM3	LSm proteins	27258
LSM4	LSm proteins	25804
LSM5	LSm proteins	23658
LSM6	LSm proteins	11157
LSM7	LSm proteins	51690
LSM8	LSm proteins	51691
ARMC7	Minor spliceosome associated proteins	79637
CRIPT	Minor spliceosome associated proteins	9419
PPIL2	Minor spliceosome associated proteins	23759
RBM48	Minor spliceosome associated proteins	84060
SCNM1	Minor spliceosome associated proteins	79005
RNPC3	Minor spliceosome specific	55599
SNRNP25	Minor spliceosome specific	79622
SNRNP35	Minor spliceosome specific	11066
SNRNP48	Minor spliceosome specific	154007
ZCRB1	Minor spliceosome specific	85437
ZMAT5	Minor spliceosome specific	55954
COIL	Other proteins involved in snRNP biogenesis	8161
SMNDC1	Other proteins involved in snRNP biogenesis	10285
SNUPN	Other proteins involved in snRNP biogenesis	10073
USB1	Other proteins involved in snRNP biogenesis	79650
AAR2	Other spliceosome associated proteins	25980
C7orf55-LUC7L2	Other spliceosome associated proteins	100996928
CELF1	Other spliceosome associated proteins	10658
CELF2	Other spliceosome associated proteins	10659
CELF3	Other spliceosome associated proteins	11189
CELF4	Other spliceosome associated proteins	56853
CELF5	Other spliceosome associated proteins	60680
CELF6	Other spliceosome associated proteins	60677
CLK1	Other spliceosome associated proteins	1195
CLK2	Other spliceosome associated proteins	1196
CLK3	Other spliceosome associated proteins	1198
CLK4	Other spliceosome associated proteins	57396
CWC25	Other spliceosome associated proteins	54883
DLP	Other spliceosome associated proteins	54957
DNAJC13	Other spliceosome associated proteins	23317
DNAJC6	Other spliceosome associated proteins	9829
DNAJC8	Other spliceosome associated proteins	22826
ELAVL2	Other spliceosome associated proteins	1993
ELAVL3	Other spliceosome associated proteins	1995
ELAVL4	Other spliceosome associated proteins	1996

ESRP1	Other spliceosome associated proteins	54845
ESRP2	Other spliceosome associated proteins	80004
EWSR1	Other spliceosome associated proteins	2130
FUBP1	Other spliceosome associated proteins	8880
FUBP3	Other spliceosome associated proteins	8939
KHSRP	Other spliceosome associated proteins	8570
LUC7L2	Other spliceosome associated proteins	51631
MATR3	Other spliceosome associated proteins	9782
NONO	Other spliceosome associated proteins	4841
NRIP2	Other spliceosome associated proteins	83714
NSRP1	Other spliceosome associated proteins	84081
PAXBP1	Other spliceosome associated proteins	94104
PPIA	Other spliceosome associated proteins	5478
PPP1CA	Other spliceosome associated proteins	5499
PPP1CB	Other spliceosome associated proteins	5500
PPP1CC	Other spliceosome associated proteins	5501
PPP1R8	Other spliceosome associated proteins	5511
PRMT5	Other spliceosome associated proteins	10419
PRPF39	Other spliceosome associated proteins	55015
PRPF4B	Other spliceosome associated proteins	8899
PTBP1	Other spliceosome associated proteins	5725
PTBP2	Other spliceosome associated proteins	58155
PTBP3	Other spliceosome associated proteins	9991
SFPQ	Other spliceosome associated proteins	6421
SRP54	Other spliceosome associated proteins	6729
SRPK1	Other spliceosome associated proteins	6732
SRPK2	Other spliceosome associated proteins	6733
SRRP35	Other spliceosome associated proteins	135295
WBP4	Other spliceosome associated proteins	11193
WDR77	Other spliceosome associated proteins	79084
XRCC6	Other spliceosome associated proteins	2547
CWC27	PPIases	10283
PPIG	PPIases	9360
PPIL3	PPIases	53938
PPIL4	PPIases	85313
PPWD1	PPIases	23398
BCAS2	Prp19 complex	10286
CDC5L	Prp19 complex	988
CTNNBL1	Prp19 complex	56259
CWC15	Prp19 complex	51503
HSPA1A	Prp19 complex	3303
HSPA1B	Prp19 complex	3304
HSPA1L	Prp19 complex	3305
HSPA2	Prp19 complex	3306
HSPA6	Prp19 complex	3310
HSPA8	Prp19 complex	3312
PLRG1	Prp19 complex	5356
PQBP1	Prp19 complex	10084
PRPF19	Prp19 complex	27339
WBP11	Prp19 complex	51729

AQR	Prp19 related	9716
BUD31	Prp19 related	8896
CCDC12	Prp19 related	151903
CRNKL1	Prp19 related	51340
ISY1	Prp19 related	57461
ISY1-RAB43	Prp19 related	100534599
PPIE	Prp19 related	10450
PPIL1	Prp19 related	51645
PRCC	Prp19 related	5546
RBM22	Prp19 related	55696
SNW1	Prp19 related	22938
SYF2	Prp19 related	25949
XAB2	Prp19 related	56949
BUD13	RES complex	84811
RBMX2	RES complex	51634
SNIP1	RES complex	79753
ADAR	RNA binding proteins	103
ADARB1	RNA binding proteins	104
ADARB2	RNA binding proteins	105
CBX3	RNA binding proteins	11335
CIRBP	RNA binding proteins	1153
CSTF2	RNA binding proteins	1478
DPF2	RNA binding proteins	5977
IGF2BP1	RNA binding proteins	10642
IGF2BP3	RNA binding proteins	10643
KHDRBS1	RNA binding proteins	10657
KHDRBS2	RNA binding proteins	202559
KHDRBS3	RNA binding proteins	10656
MBNL1	RNA binding proteins	4154
MBNL2	RNA binding proteins	10150
MBNL3	RNA binding proteins	55796
NOVA1	RNA binding proteins	4857
NOVA2	RNA binding proteins	4858
QKI	RNA binding proteins	9444
RBFOX1	RNA binding proteins	54715
RBFOX2	RNA binding proteins	23543
RBFOX3	RNA binding proteins	146713
RBM12	RNA binding proteins	10137
RBM12B	RNA binding proteins	389677
RBM14	RNA binding proteins	10432
RBM15	RNA binding proteins	64783
RBM15B	RNA binding proteins	29890
RBM18	RNA binding proteins	92400
RBM26	RNA binding proteins	64062
RBM27	RNA binding proteins	54439
RBM3	RNA binding proteins	5935
RBM4	RNA binding proteins	5936
RBM4B	RNA binding proteins	83759
RBM7	RNA binding proteins	10179
RNPS1	RNA binding proteins	10921

SCAF8	RNA binding proteins	22828
SERBP1	RNA binding proteins	26135
STRBP	RNA binding proteins	55342
TIA1	RNA binding proteins	7072
TIAL1	RNA binding proteins	7073
TOE1	RNA binding proteins	114034
YBX3	RNA binding proteins	8531
YTHDC1	RNA binding proteins	91746
ZFR	RNA binding proteins	51663
ZFR2	RNA binding proteins	23217
ZNF326	RNA binding proteins	284695
DDX1	RNA helicase like proteins	1653
DDX17	RNA helicase like proteins	10521
DDX18	RNA helicase like proteins	8886
DDX26B	RNA helicase like proteins	203522
DDX27	RNA helicase like proteins	55661
DDX39A	RNA helicase like proteins	10212
DDX3X	RNA helicase like proteins	1654
DDX3Y	RNA helicase like proteins	8653
DDX43	RNA helicase like proteins	55510
DDX50	RNA helicase like proteins	79009
DDX52	RNA helicase like proteins	11056
DHX30	RNA helicase like proteins	22907
DHX9	RNA helicase like proteins	1660
DQX1	RNA helicase like proteins	165545
ERH	RNA helicase like proteins	2079
SNRPB	Sm proteins	6628
SNRPD1	Sm proteins	6632
SNRPD2	Sm proteins	6633
SNRPD3	Sm proteins	6634
SNRPE	Sm proteins	6635
SNRPF	Sm proteins	6636
SNRPG	Sm proteins	6637
DDX20	SMN complex	11218
GEMIN2	SMN complex	8487
GEMIN4	SMN complex	50628
GEMIN5	SMN complex	25929
GEMIN6	SMN complex	79833
GEMIN7	SMN complex	79760
GEMIN8	SMN complex	54960
SMN1	SMN complex	6606
SMN2	SMN complex	6607
STRAP	SMN complex	11171
PCBP3	Spliceosome associated proteins	54039
PCBP4	Spliceosome associated proteins	57060
AKAP17A	SR proteins	8227
CLASRP	SR proteins	11129
PNISR	SR proteins	25957
SCAF4	SR proteins	57466
SREK1	SR proteins	140890

SRSF1	SR proteins	6426
SRSF10	SR proteins	10772
SRSF11	SR proteins	9295
SRSF2	SR proteins	6427
SRSF3	SR proteins	6428
SRSF4	SR proteins	6429
SRSF5	SR proteins	6430
SRSF6	SR proteins	6431
SRSF7	SR proteins	6432
SRSF8	SR proteins	10929
SRSF9	SR proteins	8683
SUGP2	SR proteins	10147
TRA2A	SR proteins	29896
TRA2B	SR proteins	6434
ARGLU1	SR-related proteins	55082
SON	SR-related proteins	6651
SRRM1	SR-related proteins	10250
SRRM2	SR-related proteins	23524
CDC40	STEP II	51362
DHX38	STEP II	9785
DHX8	STEP II	1659
PRPF18	STEP II	8559
RSRC1	STEP II	51319
SLU7	STEP II	10569
THOC5	TREX complex	8563
THOC6	TREX complex	79228
THOC7	TREX complex	80145
DDX5	U1 snRNP related	1655
PRPF40A	U1 snRNP related	55660
PRPF40B	U1 snRNP related	25766
RBM25	U1 snRNP related	58517
TCERG1	U1 snRNP related	10915
SNRNP70	U1 snRNP specific	6625
SNRPA	U1 snRNP specific	6626
SNRPC	U1 snRNP specific	6631
CHERP	U2 snRNP related	10523
DDX46	U2 snRNP related	9879
DHX15	U2 snRNP related	1665
PUF60	U2 snRNP related	22827
RBM17	U2 snRNP related	84991
RP9	U2 snRNP related	6100
U2AF1	U2 snRNP related	7307
U2AF1L4	U2 snRNP related	199746
U2AF1L5	U2 snRNP related	102724594
U2AF2	U2 snRNP related	11338
U2SURP	U2 snRNP related	23350
ZRSR2	U2 snRNP related	8233
DDX42	U2 snRNP specific	11325
PHF5A	U2 snRNP specific	84844
SF3A1	U2 snRNP specific	10291

SF3A2	U2 snRNP specific	8175
SF3A3	U2 snRNP specific	10946
SF3B1	U2 snRNP specific	23451
SF3B2	U2 snRNP specific	10992
SF3B3	U2 snRNP specific	23450
SF3B4	U2 snRNP specific	10262
SF3B5	U2 snRNP specific	83443
SF3B6	U2 snRNP specific	51639
SNRPA1	U2 snRNP specific	6627
SNRPB2	U2 snRNP specific	6629
NHP2L1	U4/U6 snRNP specific	4809
PPIH	U4/U6 snRNP specific	10465
PRPF3	U4/U6 snRNP specific	9129
PRPF31	U4/U6 snRNP specific	26121
PRPF4	U4/U6 snRNP specific	9128
SART3	U4/U6 snRNP specific	9733
CWC22	U4/U6.U5 tri-snRNP related	57703
DHX16	U4/U6.U5 tri-snRNP related	8449
GPKOW	U4/U6.U5 tri-snRNP related	27238
HSPB1	U4/U6.U5 tri-snRNP related	3315
KIN	U4/U6.U5 tri-snRNP related	22944
PRPF38A	U4/U6.U5 tri-snRNP related	84950
PRPF38B	U4/U6.U5 tri-snRNP related	55119
SKIV2L2	U4/U6.U5 tri-snRNP related	23517
ZMAT2	U4/U6.U5 tri-snRNP related	153527
ZNF830	U4/U6.U5 tri-snRNP related	91603
SART1	U4/U6.U5 tri-snRNP specific	9092
SNRNP27	U4/U6.U5 tri-snRNP specific	11017
USP39	U4/U6.U5 tri-snRNP specific	10713
CD2BP2	U5 snRNP specific	10421
DDX23	U5 snRNP specific	9416
EFTUD2	U5 snRNP specific	9343
PRPF6	U5 snRNP specific	24148
PRPF8	U5 snRNP specific	10594
SNRNP200	U5 snRNP specific	23020
SNRNP40	U5 snRNP specific	9410
TXNL4A	U5 snRNP specific	10907
